# Performance optimization design for constructed wetland coupled with microbial fuel cell for rural domestic sewage treatment

**DOI:** 10.1371/journal.pone.0350011

**Published:** 2026-05-27

**Authors:** Tuodi Zhang, Songtao Shen, Yingyi Xu

**Affiliations:** 1 School of Environment and Resource, Southwest University of Science and Technology, Mianyang, China; 2 School of Culture and Education, Tianfu College of Southwestern University of Finance and Economics, Mianyang, China; Indian Institute of Technology Delhi, INDIA

## Abstract

A vertical-flow constructed wetland-microbial fuel cell (CW-MFC) was established with *Acorus calamus* as the wetland vegetation for rural domestic wastewater treatment under low-temperature winter conditions. Response surface methodology (RSM) coupled with central composite design (CCD) was used to optimize three key electrode parameters: electrode plate projection coefficient (PC), inter-electrode distance (ID), and external resistance (ER), and to evaluate their effects on COD removal efficiency. Compared with the standalone constructed wetland (CW), the CW-MFC system significantly improved wastewater treatment performance: the effluent COD concentration of the CW was 66.14 mg/L, exceeding the first-class discharge limit (60.00 mg/L) specified in the “Water Pollutant Discharge Standard for Rural Domestic Sewage Treatment Facilities” (DB51/2626–2019). In contrast, the effluent COD concentrations of the CW-MFC system ranged from 18.81 to 54.06 mg/L, all meeting the aforementioned first-class standard, with a significantly higher COD removal rate than the CW (P < 0.05). After electrode parameter optimization, the optimal configuration was determined as PC = 0.33, ID = 272.94 mm, and ER = 1619.31 Ω. Under these optimized conditions, the CW-MFC system achieved a COD removal efficiency of 89.14%, which was consistent with the model-predicted value (88.06%) with a deviation of <1.2%. This confirmed that electrode parameter optimization effectively enhanced the treatment performance of the CW-MFC system. These findings could contribute new perspectives to the performance optimization of CW-MFC systems applied to decentralized rural domestic sewage treatment, particularly under low-temperature winter scenarios.

## Introduction

Following the establishment and operational commencement of centralized sewage treatment facilities in China’s urban zones, decentralized domestic wastewater in rural regions has increasingly become a critical contributor to aquatic contamination. This persistent environmental challenge has impeded the sustained enhancement of national water quality, particularly manifesting in the ecologically fragile and economically underdeveloped western territories [1]. Rural domestic sewage exhibits characteristics such as decentralized pollution sources, intricate collection processes, small-scale treatment, and frequent variations in both water quantity and quality [2]. Consequently, building large-scale centralized wastewater treatment plants with substantial investments is impractical, rendering decentralized treatment the preferred solution [3]. Constructed wetland (CW) is a quintessential ecological treatment technology, extensively utilized in rural regions of China for wastewater treatment owing to its benefits of simple structure, straightforward operation, and minimal energy consumption [4]. CWs accomplish contaminant removal through synergistic interactions of substrate filtration, plant assimilation, and microbial metabolism. This integrated purification mechanism exhibits pronounced seasonal variability due to temperature-dependent biological processes [5]. During winter conditions with sustained low temperatures, vegetation enters dormancy with reduced nutrient uptake capacity, while psychrophilic microbial communities demonstrate limited enzymatic activity, collectively leading to a marked decline in system-wide decontamination efficiency [6]. In recent years, the advancement of microbial fuel cell (MFC) technology has driven the emergence of a coupled system to enhance wastewater treatment efficiency of CWs [7].

**Fig 1:**
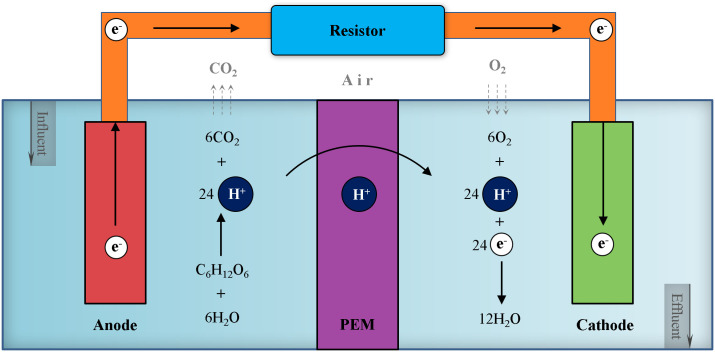
Schematic diagram of pollutant removal mechanism in the microbial fuel cell (MFC).

The principle of pollutant removal in the MFC was illustrated in Fig 1. In this system, electroactive microorganisms (predominantly exoelectrogenic bacteria, such as *Shewanella* and *Geobacter*) residing in the anode chamber decompose organic matter present in wastewater under anaerobic conditions; this decomposition process yields carbon dioxide, protons, and electrons, where exoelectrogenic bacteria play a crucial role in transferring electrons directly to the anode without the need for electron shuttles. The protons then traverse a proton exchange membrane (PEM) from the anode chamber to the cathode chamber; simultaneously, electrons are conducted through an external circuit to the cathode, generating an electric current. The cathode chamber is aerobic, as it requires oxygen to maintain the cathodic reaction, and the main electron acceptor present in the cathode chamber is oxygen (O₂), which is a common and efficient electron acceptor in most MFC configurations. In the cathode chamber, the protons that have migrated through the PEM react with the oxygen (electron acceptor) and the electrons that have traveled through the external circuit, ultimately producing water.

In an integrated process of constructed wetland coupled with microbial fuel cell (CW-MFC) where glucose acted as an organic pollutant and oxygen served as the terminal electron acceptor, the anodic oxidation, the cathodic reduction and the overall reaction could be respectively expressed by Equation (1), (2) and (3).


$$\begin{array}{c} {\text{C}}_{\text{6}}{\text{H}}_{\text{12}}{\text{O}}_{\text{6}}\;+\;\text{6}\;{\text{H}}_{\text{2}}\text{O}\to \;\text{6}\;{\text{CO}}_{\text{2}}\;+\;{\text{24H}}^{+}\;+\;{\text{24e}}^{-} \end{array}$$ (1)

$$\begin{array}{c} \text{6}\;O_{\text{2}}\;+\;{\text{2}\text{4}H}^{+}\;+\;{\text{2}\text{4}e}^{-}\;\to \;\text{1}\text{2}\;H_{\text{2}}O \end{array}$$ (2)

$$\begin{array}{c} C_{\text{6}}H_{\text{1}\text{2}}O_{\text{6}}\;+\;\text{6}\;O_{\text{2}}\;\to \;\text{6}\;{CO}_{\text{2}}\;+\;\text{6}\;H_{\text{2}}O \end{array}$$ (3)

The CW-MFC integrates the bioelectrochemical activity of MFCs with the natural purification mechanisms of CWs, forming a structurally complementary and functionally synergistic system, thereby enhancing the effect of wastewater treatment [8]. Employing self-prepared sewage for the investigation of anaerobic ammonia oxidation, the CW-MFC system was found to exhibit enhancements of 20.0% and 13.6% in the removal efficiencies of total nitrogen and ammonia nitrogen, respectively, compared with the standalone constructed wetland system [9]. Doherty et al. investigated the phosphorus removal capability of aluminum sludge-derived CW-MFC for treating aquaculture wastewater and demonstrated that the TP removal rate was increased by 15%−20% compared with traditional CW systems [10]. Experimental results from domestic sewage treatment indicated that CW-MFC achieved a 12% higher COD removal efficiency than conventional CW [11]. Biswas et al. experimentally demonstrated that MFC improved the synthetic greywater treatment performance of CW, with COD removal efficiency enhanced by 10%−15% [12]. It was evident that MFC could effectively enhance the degradation capacity of constructed wetlands (CWs) for both organic pollutants and nutrient pollutants in sewage. However, the enhancement mechanisms and effects differed between these two types of pollutants. Specifically, the enhancement effect on degradation of organic pollutants was achieved through the direct strengthening of electrochemical reactions, whereas the improvement in nutrient pollutant degradation was an indirect consequence resulting from the increased abundance of electroactive microorganisms in the CW system [13]. Extensive research has been carried out on the application of CW-MFC integrated systems in rural sewage treatment. To achieve superior treatment performance, most previous studies have focused primarily on electrode materials, system configurations, electrogenesis performance, and the regulation of operational parameters [14–17], whereas the design optimization of key electrode parameters remains largely underexplored. To clarify the direct contribution of MFC to the pollutant removal performance of constructed wetlands CW in wastewater treatment processes, this study focused on three key electrode design parameters in MFC, namely electrode dimension, electrode distance, and external resistance. Specifically, the study aimed to explore the relationship between these electrode-related parameters and the degradation efficiency of the most directly relevant pollutants (organic pollutants) in the CW-MFC coupled system. Based on the interaction mechanisms among these three parameters, an optimal configuration scheme was further proposed to maximize the overall wastewater treatment capacity of the system.

## Materials and methods

### Experimental equipment and materials

**Fig 2:**
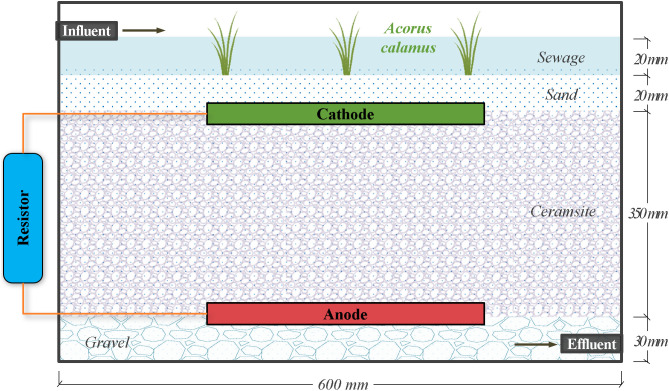
Schematic diagram of the structure and dimensions of the vertical-flow constructed wetland-microbial fuel cell (CW-MFC) reactor.

The vertical flow CW-MFC reactor was shown in Fig 2, where sewage entered from the top, exited from the bottom. The reactor was made of polypropylene, rectangular parallelepiped shaped, with the size of 600 mm × 450 mm × 450 mm. The reactor was filled with gravel, ceramic particles, and sand from bottom to top, with average particle sizes of 15 mm, 8 mm, and 0.5 mm, and filling thicknesses of 30 mm, 350 mm, and 20 mm, respectively. 10 plants of *Acorus calamus* were uniformly distributed across the sand layer surface, with the water level maintained at 20 mm above the substrate interface. The reactor had a geometric volume of 113.4 L, an effective volume of 22.7 L, and a porosity of 20%. A flexible graphite carbon felt electrode was selected and installed in the reactor owing to its simple structure, low cost, mild operating conditions, and excellent safety performance. Characterized by high electrical conductivity and a large specific surface area, this electrode was typically modified with functional groups or nanomaterials on its surface to facilitate microbial adhesion. In this study, the flexible graphite carbon felt electrode had a thickness of 1 mm and a fixed width of 250 mm, with its area regulated by varying the electrode length from 140 mm to 540 mm. [18]. The anode was fixed at a vertical distance of 30 mm from the reactor bottom, while the cathode was positioned parallel to the anode at varying intervals (200 mm, 250 mm, 300 mm, etc.). A closed circuit was established by connecting the anode and cathode to an external variable resistor via conductive wires. The control check group did not incorporate any electrodes, with only the CW system utilized for sewage treatment. 0.5 mm^2^ solid copper wires were utilized to establish electrical connections. Tin-plated solder was applied to affix the copper wires to the electrodes, and the soldered joints were sealed with epoxy resin. Additionally, cold welding was used to connect the copper wires to the external resistors, with the welded joints wrapped in electrical tape for insulation.

The CW-MFC reactor was operated continuously with a hydraulic loading rate of 0.19 m^3^/(m^2^·d), uninoculated activated sludge, and fed with pretreated wastewater obtained from the 5‑mm fine screen effluent of the sewage treatment plant at Southwest University of Science and Technology. This influent sewage, primarily derived from graduate student dormitories and surrounding self-built houses, exhibited characteristic low-flow conditions with high fluctuations in pollutant concentrations, similar to non-centralized residential wastewater in rural settings.

### Experimental procedure and analysis methods

To tackle the insufficient treatment performance of CW in winter and minimize interference from external environmental factors (e.g., temperature, light, and precipitation), experimental operations were strictly confined to non-precipitation periods during cold months. Following the stabilization of the CW-MFC process, influent and effluent samples maintained at 8 ± 0.5℃ were collected, typically around midday from November to December 2024. As the pollutant most directly influenced by the MFC to be coupled with the CW, the chemical oxygen demand (COD) was selected as the evaluation indicator for electrode parameter optimization. COD was determined using the potassium dichromate method, with the removal efficiency subsequently calculated through the following equation (4).


$$\begin{array}{c} \eta \;=\frac{C_{i}-C_{e}}{C_{i}}\times \text{100} \end{array}$$ (4)

Where *η* denoted the removal rate of COD, with *C*_*i*_ and *C*_*e*_ corresponding to the COD concentrations in influent and effluent sewages respectively.

### Experimental design and optimization method

Response Surface Methodology (RSM) represents a powerful statistical approach for addressing nonlinear data processing. Through regression analysis of process variables and subsequent visualization via three-dimensional response surfaces and two-dimensional contour plots, researchers can efficiently determine response values across different factor levels [19]. Central Composite Design (CCD) stands as a widely adopted experimental design framework within RSM, particularly effective for multistage investigative processes. This design framework provides an optimized solution for constructing second-order polynomial models, enabling comprehensive analysis of multifactorial interactions across varying parameter intensities. The rotational symmetry and experimental adaptability inherent to CCD are achieved through strategic incorporation of axial points, ensuring both spatial uniformity and procedural continuity in data acquisition [20].

Structurally, CCD employs a five-tiered coding system: 0 (central reference point), ± 1 (factorial boundaries), and ±α (axial extremes). Here, α represents a geometrically determined axial distance, typically calibrated to preserve rotational properties or orthogonality based on design objectives. This hierarchical configuration allows precise characterization of quadratic response behaviors while maintaining experimental efficiency, making CCD particularly advantageous for exploring complex nonlinear relationships with minimized experimental runs [21]. In this study, the experimental design matrix detailing operational levels and variables for the CCD is systematically organized in Table 1. The CCD framework comprised three key design parameters: PC defined as the Projection coefficient of electrode plate (dimensionless parameter), expressed by Equation (5), ID denoting inter-electrode distance (mm), and ER characterizing external circuit resistance (Ω).

**Table 1:** Coded and uncoded levels of key electrode parameters for CCD.

Coded level	Uncoded Level		
	ID (mm)	PC	ER (Ω)
−1.68	165.91	0.13	659.10
−1	200	0.2	1000
0	250	0.3	1500
1	300	0.4	2000
1.68	334.09	0.47	2340.90


$$\begin{array}{c} PC{=}^{EA}{╱}_{RA}\; \end{array}$$ (5)

Where PC represented the projection coefficient of the electrode plate, with EA and RA denoting the electrode area and reactor cross-sectional area, respectively.

A CCD with three independent variables was implemented using three operational levels, comprising a total of 20 experimental runs. The design configuration consisted of 8 star points, 6 axial points, and 6 center points, adhering to standard CCD principles. Subsequent statistical analysis of the experimental data enabled the development of a quadratic regression model correlating design parameters with COD removal efficiency. This second-order polynomial model, mathematically expressed in Equation (6), effectively integrated the three design parameters through response surface methodology.


$$\begin{array}{c} \eta =C_{\text{0}}+\sum_{i=1}^{n}C_{i}X_{i}+\sum_{i=1}^{n}C_{ii}\overset{\text{2}}{\underset{i}{X}}+\sum_{1\leq i<j\leq n}^{n}C_{ij}X_{i}X_{j} \end{array}$$ (6)

Where the dependent variable *η* denoted the removal rate of COD, while *X* represented the independent variables encompassing PC, ID and ER. The regression structure incorporated a constant term *C*_*0*_, with linear coefficients denoted by *C*_*i*_. Quadratic term coefficients and interaction term coefficients were characterized by *C*_*ii*_ and *C*_*ij*_ respectively.

## Results and discussion

### Sewage treatment performance

**Fig 3:**
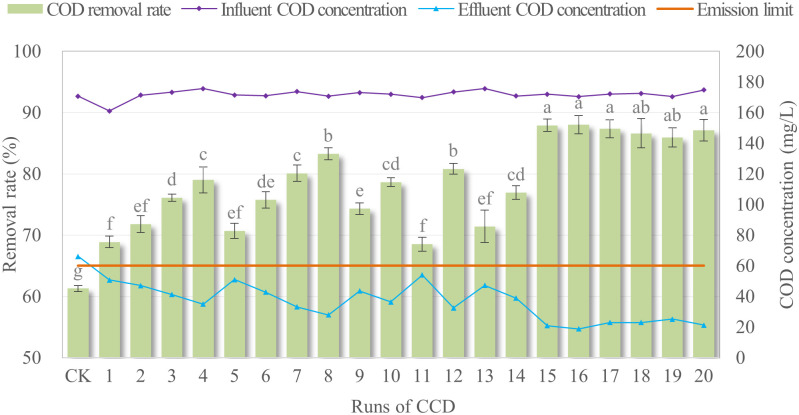
Experimental treatments for sewage influent and effluent results. CK represented the independent CW treatment; 1-20 represented the CW-MFC treatments with 20 electrode parameter configurations designed by CCD; different letters (a-g) indicated significant differences among treatments (P < 0.05).

The CCD experiments and a control check (CK) were performed to assess the COD removal efficiency, and the calculated results were summarized in Fig 3. A comparison between CW (CK) and CW-MFC treatment (runs 1–20 designed by CCD) revealed significant differences in sewage treatment efficiency. The effluent COD concentration of CK was 66.14 mg/L, which exceeded the first-class discharge limit (60.00 mg/L) specified in the “Water Pollutant Discharge Standard for Rural Domestic Sewage Treatment Facilities” (DB51/2626–2019), thus failing to meet the required discharge criteria. In contrast, all effluent COD concentrations of CW-MFC treatment ranged from 18.81 mg/L to 54.06 mg/L, all complying with the aforementioned first-class standard. The COD removal rate of CW-MFC was significantly higher than that of CW (P < 0.05), demonstrating that the introduction of MFC significantly enhanced the sewage treatment performance of the CW system.

### Model fitting

For the 20 runs of CW-MFC system designed by CCD (excluding CK, which was the blank group treated by CW alone), the mean COD removal rate exhibited a significant variation ranging from 68.53% to 88.04% (Table 2), indicating a remarkable difference of 19.51% between the maximum and minimum values. Notably, center-run configurations exhibited superior performance in organic removal efficiency, demonstrating an average COD elimination rate of 87.17%. This represented a significant 15.5% enhancement compared to alternative experimental configurations (Star and Axial arrangements), which achieved a mean removal rate of 75.45%. The experimental sequence and rotational parameters were rigorously maintained throughout the study, confirming proper implementation of the test protocol.

**Table 2:** Electrode parameter design and corresponding results for all experimental groups.

Run	Type	Uncoded level			COD removal rate (%)	Standard deviation
		ID (mm) X_1_	PC X_2_	ER(Ω) X_3_		
1	Star	200	0.2	1000	68.89	1.61
2	Star	300	0.2	1000	71.78	2.37
3	Star	200	0.4	1000	76.12	0.99
4	Star	300	0.4	1000	79.02	3.68
5	Star	200	0.2	2000	70.70	2.17
6	Star	300	0.2	2000	75.76	2.34
7	Star	200	0.4	2000	80.11	2.31
8	Star	300	0.4	2000	83.27	1.73
9	Axial	165.91	0.3	1500	74.32	1.65
10	Axial	334.09	0.3	1500	78.66	1.21
11	Axial	250	0.131821	1500	68.53	1.97
12	Axial	250	0.468179	1500	80.83	1.55
13	Axial	250	0.3	659.104	71.42	4.57
14	Axial	250	0.3	2340.9	76.93	1.93
15	Center	250	0.3	1500	87.93	1.79
16	Center	250	0.3	1500	88.04	2.58
17	Center	250	0.3	1500	87.36	2.53
18	Center	250	0.3	1500	86.63	4.13
19	Center	250	0.3	1500	85.96	2.70
20	Center	250	0.3	1500	87.11	3.05

Using Design-Expert 10.0.8.0, a predictive quadratic polynomial regression model was developed with coefficients derived through the least squares method. The model incorporates unencoded numerical values representing the constant term, linear coefficient, quadratic term coefficient, and interaction coefficient, which collectively form the following predictive equation (7).


$$\begin{array}{cc} \eta \;= & \;\;-\text{97}.\text{07 }+\;{\text{0}.\text{73}X}_{\text{1}}+\;{\text{289}.\text{24}X}_{\text{2}}\;+\;{\text{0}.\text{05}X}_{\text{3}}\;-\;{\text{0}.\text{05}X}_{\text{1}}\cdot X_{\text{2}}\;+\text{1}.\text{22}\times {\text{10}}^{-\text{5}}\;X_{\text{1}}\cdot X_{\text{3}}\;+\;\text{6}.\text{10}\times {\text{10}}^{-\text{3}}\;X_{\text{2}}\cdot X_{\text{3}}\;\\ & \;-\;\text{1}.\text{40}\times {\text{10}}^{-\text{3}}\overset{\text{2}}{\underset{\text{1}}{X}}\;-\;\overset{\text{2}}{\underset{\text{2}}{\text{413}.\text{91}X}}\;-\;\text{1}.\text{73}\times {\text{10}}^{-\text{5}}\overset{\text{2}}{\underset{\text{3}}{X}} \end{array}$$ (7)

In the equation, *η* represented the COD removal rate, while *X*_*2*_ denoted PC. The variables *X*_*1*_ and *X*_*3*_ corresponded to ID and ER, respectively.

The quadratic polynomial regression model for predicting the COD removal rate (Equation 6) was evaluated for its goodness of fit through analysis of variance (ANOVA), with the results summarized in Table 3. The polynomial regression analysis identified statistically significant effects (P < 0.05) for both linear terms (*X*_*1*_, *X*_2_, *X*_3_) and quadratic terms (*X*_*1*_^*2*^, *X*_2_^*2*^, *X*_3_^*2*^) of the independent variables on COD removal efficiency, whereas all pairwise interaction effects (*X*_*1*_ *X*_2_, *X*_1_ *X*_3_, *X*_*2*_*X*_3_) failed to demonstrate significant process impacts.

**Table 3:** The variance analysis of the fitting model.

Source	Sum of Squares	DF	Mean Square	*F* Value	*P* Value
Model	852.20	9	94.69	93.32	< 0.0001
X_1_	33.29	1	33.29	32.81	0.0002
X_2_	198.60	1	198.60	195.74	< 0.0001
X_3_	39.73	1	39.73	39.15	< 0.0001
X_1_·X_2_	0.45	1	0.45	0.45	0.5190
X_1_·X_3_	0.74	1	0.74	0.73	0.4122
X_2_·X_3_	0.74	1	0.74	0.73	0.4122
X_1_^2^	176.48	1	176.48	173.94	< 0.0001
X_2_^2^	246.90	1	246.90	243.34	< 0.0001
X_3_^2^	268.42	1	268.42	264.55	< 0.0001
Residual	10.15	10	1.01		
Lack of fit	7.03	5	1.41	2.25	0.1966
Pure error	3.12	5	0.62		
Cor Total	862.35	19			

In ANOVA, the *F*-value represents the ratio of the between-group mean square to the within-group mean square. This statistical measure, at a specified confidence level, helps determine whether observed differences between datasets originate from systematic variations or random errors. Both the *F*-value and *p*-value in the model indicate the statistical significance of each control factor’s influence. Specifically, higher *F*-values coupled with smaller *p*-values (typically <0.05) demonstrate stronger evidence against the null hypothesis, reflecting more significant correlations between variables [22]. The regression model demonstrated exceptional statistical significance with an *F*-value of 93.32 and a *p*-value < 0.0001, confirming excellent model adequacy across the experimental domain. Notably, the lack-of-fit term showed non-significance (*p* = 0.1966), validating model specification. The coefficient of determination (*R*^*2*^ = 0.9882) revealed that 98.82% of response variability could be explained by the model parameters, leaving only 1.18% unexplained variance. Furthermore, the model exhibited remarkable precision as evidenced by a coefficient of variation (*CV* = 1.28%) well below the 10% acceptability threshold, ensuring the reliability of central composite design experimental outcomes [23].The signal-to-noise ratio quantified by adequate precision (26.2) demonstrated exceptional measurement reliability, greatly exceeding the recommended threshold of 4.0, thereby confirming the high reproducibility of central composite design experimental outputs [24].

In regression analysis, residuals represent the discrepancy between observed experimental outcomes and values predicted by the fitted regression model. A fundamental assumption underlying regression methodology requires that these residuals follow a normal distribution. Conducting formal normality tests on residuals serves to verify this critical statistical presumption, thereby validating model reliability and enhancing the credibility of regression conclusions. Beyond quantitative validation, residual plots provide indispensable visual diagnostics by revealing latent data patterns and detecting anomalies that numerical summaries might obscure, making them crucial components in comprehensive model evaluation [25].

**Fig 4:**
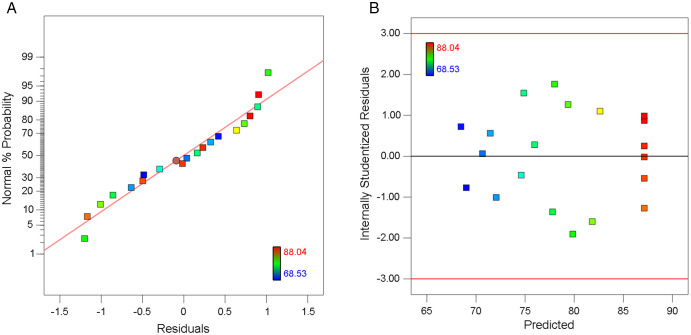
Residual distribution analysis of the quadratic regression model for COD removal efficiency prediction. **(a)** Normal probability of the residuals. **(b)** Distribution of prediction residuals.

As illustrated in Fig 4a, the quantile-quantile plot demonstrated a linear alignment between the empirical cumulative distribution of COD removal rates and the theoretical normal distribution quantiles. The close adherence of data points to the reference line statistically validated the normality assumption. The distribution of residuals plotted against predicted values demonstrated a random scattering pattern without discernible trends or systematic deviations (Fig 4b), substantiating that the residuals behaved as white noise. The residual series contained neither deterministic patterns nor extractable information that could be leveraged for model improvement, thereby validating the sufficiency of the regression model specification in capturing all systematic relationships within the dataset [26].

**Fig 5:**
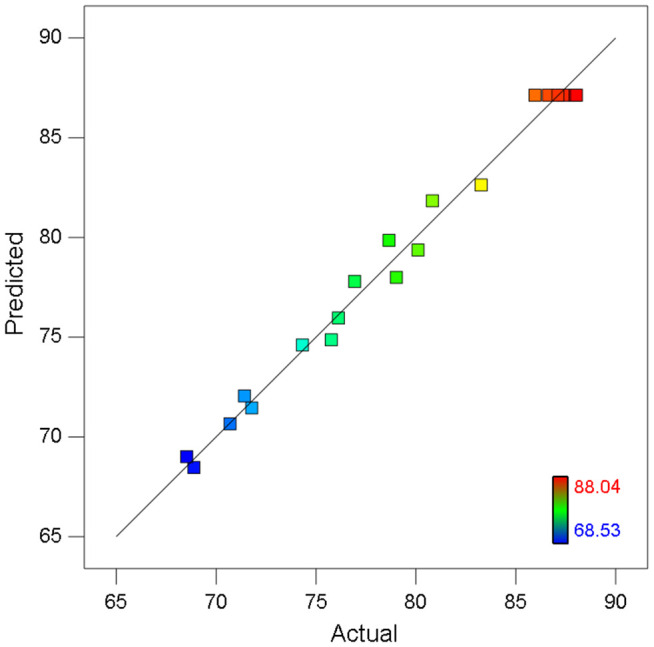
Correlation between actual and predicted COD removal rates of the CW-MFC system.

As shown in Fig 5 depicting the correlation between measured and modeled values, the data points clustered closely around the prediction reference line. This distribution pattern indicates that the experimentally observed COD removal efficiencies aligned remarkably well with the values forecasted by the regression analysis model.

### Analysis of individual factor influences

**Fig 6:**
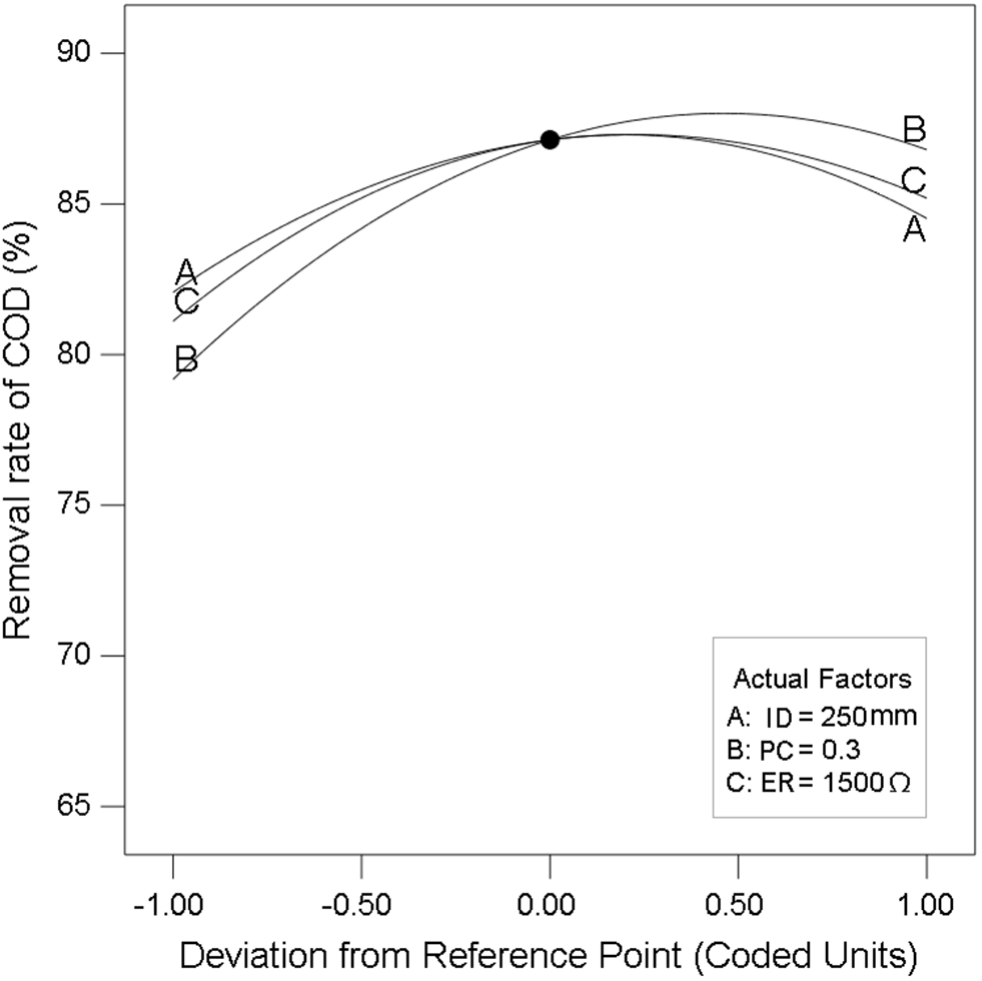
Effects of electrode plate projection coefficient (PC), inter-electrode distance (ID), and external resistance (ER) on COD removal rate of the CW-MFC system.

To facilitate comparative analysis of three contributing factors on COD removal efficiency under consistent scale conditions, Fig 6 displayed normalized curves within a unified coordinate system. The abscissa represented coded parameter variations (ID, PC, and ER) while the ordinate quantified the corresponding COD removal rate percentages. The three curves exhibited consistency throughout CCD experimental sequence, each characterized by a primary ascending phase succeeded by a controlled descent with defined peak values.

Regarding PC, increasing the electrode plate surface area enhanced the available colonization sites for electroactive microorganisms, thereby promoting microbial biomass accumulation. This enrichment of electroactive microbial communities strengthened both pollutant degradation efficiency and bioelectricity generation through intensified extracellular electron transfer and metabolic activity. However, excessive electrode dimensions impeded plant root penetration, suppressed oxygen secretion from plant rhizosphere systems, and obstructed oxygen diffusion into the cathode compartment. These combined effects reduced the activity of aerobic microorganisms and compromise overall purification performance [27]. Similarly, an ID below the critical threshold fails to establish an effective potential gradient between electrodes, resulting in diminished bioelectricity output [28]. Conversely, excessive ID elevated internal resistance, causing electron loss and decelerated extracellular electron transfer, both scenarios ultimately suppressing power generation. This performance decline further impaired the metabolic activity of exoelectrogenic microorganisms, thereby compromising organic pollutant degradation efficiency [29]. As for ER, an excessively low external resistance accelerated electron flux beyond the critical transport rate, disrupting continuous electron transfer between electrodes and impairing both bioelectricity generation and pollutant degradation efficiency. Conversely, excessively high external resistance induced anode potential reduction, which destabilizes the thermodynamic driving force for electron transfer, ultimately suppressing system power output and catalytic oxidation capacity [30]. PC demonstrated the strongest correlation with COD removal among the three parameters, as it directly regulated the abundance of electrochemically active microorganisms [31]. Meanwhile, ID and ER primarily influenced the electron transfer kinetics, with their effects on COD removal being primarily manifested in the reaction rate [29,30]. These findings collectively confirmed the validity of the theoretical design assumptions proposed in this study, while empirically demonstrating the presence of definable optimal operating thresholds across all three design parameters.

### Interactive effects of three factors

**Fig 7:**
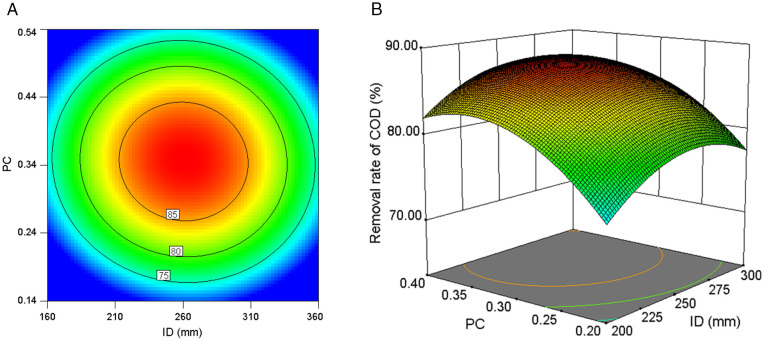
The interaction effect of ID and PC on COD removal rate at a fixed ER (1500 Ω). **(a)** Contour plot. **(b)** 3D response surface plot.

The contour plot and three-dimensional response surface plot serve as effective visual tools to graphically illustrate how interactive effects between experimental variables influence the target response metric [32]. As shown in Fig 7, the combined effect of ID and PC on COD removal efficiency became statistically significant under the central-value ER condition.

The contour lines corresponding to different COD removal rates were uniformly spaced (Fig 7a), exhibiting a concentric circular structure. The variation magnitude of COD removal efficiency induced by PC adjustments demonstrates consistency across different ID conditions, with analogous uniformity in ID-induced COD fluctuation ranges under varying PC configurations. This mutual invariance pattern suggests statistically independent regulatory pathways between PC and ID parameters, accompanied by negligible synergistic interaction effects. PC dictated the number of microbial attachment sites and consequently influenced the quantity of electrochemically active microorganisms present [31]. ID determined the electrode potential difference and thereby affected the electron transfer rate [29]. These two factors operated relatively independently during pollutant degradation, with minimal interaction between them. The surface curvature exhibited significant discrepancies along distinct coordinate axes, as illustrated in Fig 7b. The PC-axis exhibited a wider removal rate variation range compared to the ID-axis within the same unit range, demonstrating PC’s stronger influence on COD removal efficiency.

**Fig 8:**
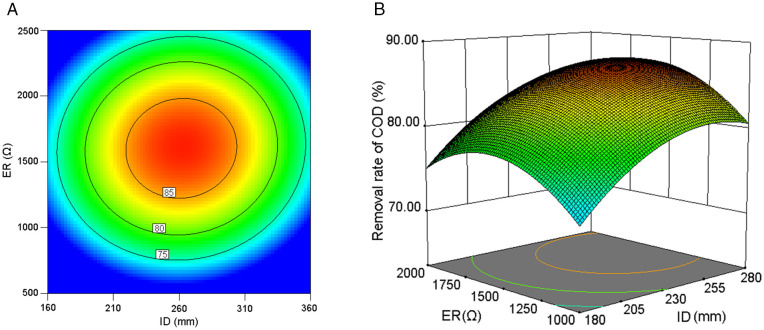
The interaction effect of ID and ER on COD removal rate at a fixed PC (0.3). **(a)** Contour plot. **(b)** 3D response surface plot.

A comparable interaction pattern between ID and ER regarding COD removal rate was observed under the central parameter condition of PC (Fig 8). The contour lines representing distinct COD removal rates maintained consistent spatial intervals (Fig 8a), manifesting a radially symmetric configuration; in parallel, this reciprocal invariance characteristic implies statistically autonomous control mechanisms between ER and ID parameters, with minimal cross-regulatory influences observed. Notably, the effects of ER and ID operated through distinct mechanisms. Unlike ID, ER exerted its influence by modulating the external electron transfer rate and anode potential, consequently impacting degradation efficiency [30]. In the response surface plot (Fig 8b), the curvature of the surface along the ID axial direction slightly exceeded that of the ER axis under normalized comparative scales, indicating a marginally stronger influence of ID on COD removal efficiency compared to ER.

**Fig 9:**
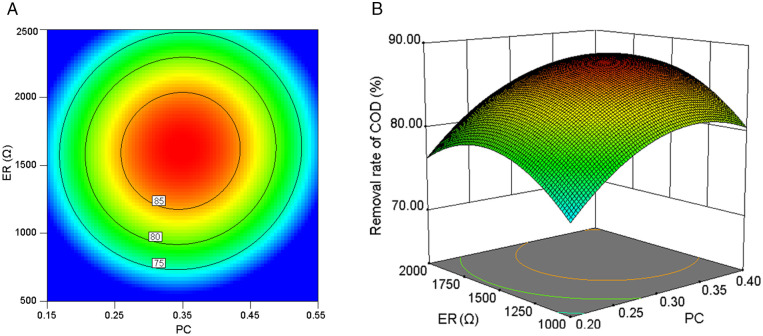
The interaction effect of PC and ER on COD removal rate at a fixed ID (250 mm). **(a)** Contour plot. **(b)** 3D response surface plot.

Under the central-value ID condition, the integrated effect of ER and PC on COD removal efficiency was demonstrated in Fig 9. The contour lines of COD removal rate determined by different ERs and PCs similarly exhibit concentric circular patterns (Fig 9a), also indicating no significant interactive effects between PC and ER. Consistent with prior analysis, the two factors exhibited distinct functional roles in pollutant degradation, with no significant interaction observed between them. As delineated in Fig 9b, the PC-axis displayed a broader variation range in removal rates relative to the ER-axis within equivalent unit intervals, indicating PC exerted a more pronounced influence on COD removal efficiency.

The optimal conditions for COD removal were determined through mathematical modeling, with the following parameters derived from regression analysis: PC of 0.33, ID of 272.94 mm, and ER of 1619.31 Ω (Table 4). To validate the predictive reliability of the model, triplicate verification experiments were systematically conducted in the constructed CW-MFC reactor under these optimized conditions. Experimental results demonstrated a mean COD removal efficiency of 89.14%, showing no significantly different from the model’s predicted value of 88.06%. The high consistency observed between experimental data and theoretical forecasts confirmed that the model was effective in optimizing electrode configuration for wastewater treatment in CW-MFC systems. The optimal electrode configuration identified in this study can exhibit considerable feasibility for scaling up to large-scale rural applications, both in newly constructed CW-MFC systems and the retrofitting of existing CWs with MFC modules. The selected flexible graphite carbon felt electrodes will possess advantages of low cost, simple structure, and high safety, which are highly compatible with the decentralized, low-investment, and easy-operation requirements of rural sewage treatment. Even for the CW that has already been constructed, the installation of parallel electrodes is feasible for the removal of blockages during cleaning procedures, without significantly altering the original substrate structure and vegetation distribution. The optimized PC, ID, and ER parameters can be scaled proportionally according to the volume and treatment capacity of large-scale rural CW-MFC reactors, ensuring that the synergistic effect between the bioelectrochemical activity of MFC and the natural purification of CW is maintained. This strategy can effectively address the problem of reduced decontamination efficiency of standalone CWs in winter. However, scalability should also consider rural practical conditions, such as adjusting electrode dimensions to avoid impeding the growth of wetland vegetation and ensuring the availability of low-cost external resistors and conductive materials in rural areas. In terms of long-term operation, the CW-MFC system may face a decline in treatment capacity due to electrode fouling, particularly the interference from plant root growth. During long-term operation, the continuous growth and proliferation of plant roots can physically encircle the electrodes, blocking the contact between electroactive microorganisms and the electrode surface, and impeding oxygen diffusion toward the cathode. Additionally, the accumulation of microbial biofilm and deposits on the electrode surface may increase internal resistance, reduce extracellular electron transfer efficiency, and further weaken the synergistic purification effect of CW and MFC, ultimately leading to a decrease in COD removal efficiency and system treatment performance. Therefore, long-term operation of scaled-up CW-MFC systems in rural areas requires regular maintenance strategies, such as periodic electrode cleaning or replacement, to mitigate the adverse effects of root interference and electrode fouling.

**Table 4:** Optimal solution and validation of the optimization model.

Optimum conditions			AN Removal rate (%)	
ID (mm)	PC	ER(Ω)	Experimental	Predicted
272.94	0.33	1619.31	89.14	88.06

## Conclusion

To address the problem of reduced decontamination efficiency of standalone CW in winter and optimize the treatment performance of the CW-MFC system for rural domestic sewage, this study conducted experiments under strictly controlled conditions: non-precipitation periods in cold months (November to December 2024), with influent and effluent samples maintained at a constant temperature of 8 ± 0.5℃. RSM combined with CCD was employed to optimize three key electrode design parameters (PC; ID and ER) and analyze their effects on COD removal efficiency, the most direct pollutant affected by MFC coupling.

The results demonstrated that the introduction of MFC significantly enhanced the sewage treatment performance of the CW system. Under the aforementioned winter experimental conditions, the standalone CW system (control check, CK) exhibited an effluent COD concentration of 66.14 mg/L, which exceeded the first-class discharge limit (60.00 mg/L) specified in the “Water Pollutant Discharge Standard for Rural Domestic Sewage Treatment Facilities” (DB51/2626–2019). In contrast, the effluent COD concentrations of the CW-MFC system ranged from 18.81 mg/L to 54.06 mg/L, all complying with the aforementioned first-class discharge standard. Additionally, the COD removal rate of CW-MFC was significantly higher than that of CW (P < 0.05), confirming the enhancement effect of MFC on CW.

Furthermore, the optimization of electrode parameters effectively improved the treatment performance of the CW-MFC system. A quadratic regression model was established with PC, ID, and ER as independent variables and COD removal efficiency as the response variable. This model exhibited high reliability, with a coefficient of determination (R² = 0.9882), significant statistical significance (P < 0.0001), and non-significant lack-of-fit (P = 0.1966), indicating that 98.82% of the variation in COD removal efficiency could be explained by the three electrode parameters. The optimal electrode configuration was determined as PC = 0.33, ID = 272.94 mm, and ER = 1619.31 Ω. Under these optimized conditions, the CW-MFC system achieved a COD removal efficiency of 89.14%, which was consistent with the model-predicted value (88.06%) with a deviation of less than 1.2%. This verifies that the optimization of electrode parameters can effectively enhance the pollutant removal capacity of the CW-MFC system. This study provides a cost-effective optimization strategy and technical support for the application of CW-MFC systems in decentralized rural domestic sewage treatment, especially under low-temperature winter conditions.

Limitations of this study include the focus on only COD as the water quality indicator and the lack of investigation on MFC-related electrical performance and energy-saving indicators; future research will expand water quality monitoring to include nutrient pollutants, strengthen the study of electrical performance and energy-saving benefits, and conduct long-term operation and scale-up tests to promote the practical application of CW-MFC technology.

## Supporting information

S1 Fig Schematic diagram of the experimental setup in this paper. (a) Leak-proof reactor casing; (b) flexible graphite carbon felt electrode; (c) Continuous water feeding device for domestic sewage; (d) The pilot-scale reactor of CW-MFC.
(DOCX)

S2 Table The original experimental data of this study. (DOCX)
