# Supplementary material for: Performance optimization design for constructed wetland coupled with microbial fuel cell for rural domestic sewage treatment
Source: PLoS One. 2026 May 27;21(5):e0350011. doi: 10.1371/journal.pone.0350011 (PMC13215525; doi:10.1371/journal.pone.0350011)
Supplement: S1 Fig — (a) Leak-proof reactor casing; (b) flexible graphite carbon felt electrode; (c) Continuous water feeding device for domestic sewage; (d) The pilot-scale reactor of CW-MFC. (DOCX) [file pone.0350011.s001.docx]

| 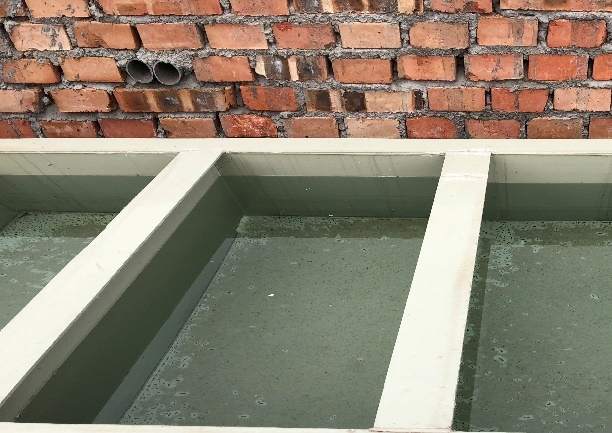 | | 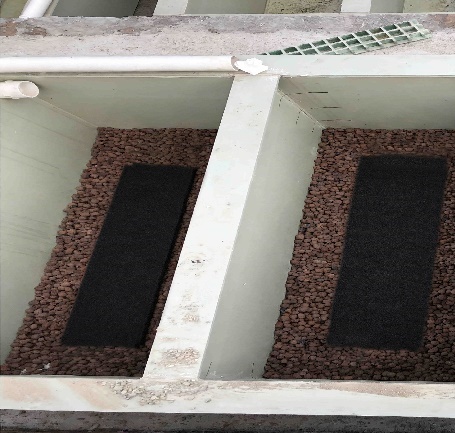 |
| --- | --- | --- |
| (a) | | (b) |
| 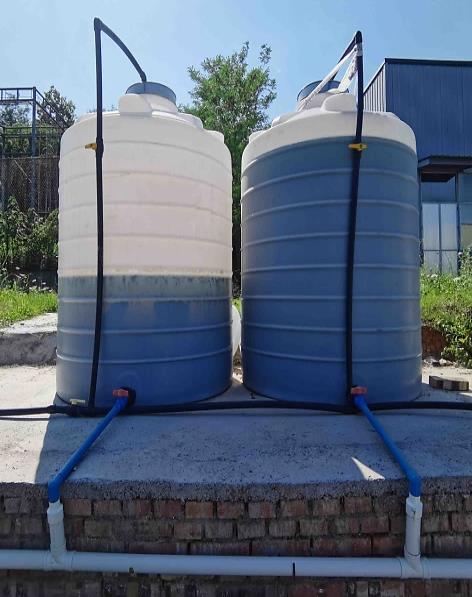 | 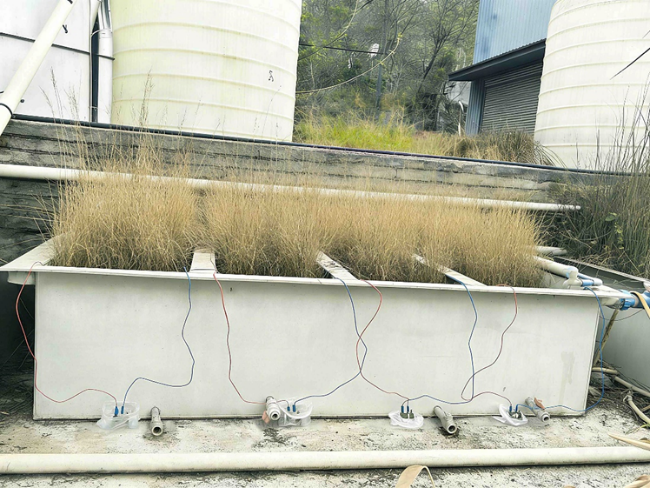 | |
| (c) | (d) | |

**Figure. Schematic diagram of the experimental setup in this paper.** (a) Leak-proof reactor casing; (b) flexible graphite carbon felt electrode; (c) Continuous water feeding device for domestic sewage; (d) The pilot-scale reactor of CW-MFC
