# Supplementary material for: Performance optimization design for constructed wetland coupled with microbial fuel cell for rural domestic sewage treatment
Source: PLoS One. 2026 May 27;21(5):e0350011. doi: 10.1371/journal.pone.0350011 (PMC13215525; doi:10.1371/journal.pone.0350011)
Supplement: S2 Table — (DOCX) [file pone.0350011.s002.docx]

**Table. The original experimental data of this study**

| RUN | Electrode configuration parameters | | | Influent COD concentration | | | Effluent COD concentration | | | COD removal rate | | | | standard error | standard deviation |
| --- | --- | --- | --- | --- | --- | --- | --- | --- | --- | --- | --- | --- | --- | --- | --- |
|  | ID (mm) | PC | ER(Ω) | #1 | #2 | #3 | #1 | #2 | #3 | #1 | #2 | #3 | average |  |  |
| CK | / | / | / | 174.49 | 168.44 | 169.37 | 66.20 | 66.65 | 65.58 | 62.06 | 60.43 | 61.35 | 61.28 | 0.4718 | 0.8173 |
| 1 | 200 | 0.2 | 1000 | 161.18 | 160.57 | 161.52 | 53.06 | 49.07 | 50.25 | 67.08 | 69.44 | 70.15 | 68.89 | 0.9279 | 1.6072 |
| 2 | 300 | 0.2 | 1000 | 176.31 | 167.11 | 170.66 | 45.26 | 47.86 | 48.16 | 74.33 | 71.36 | 69.65 | 71.78 | 1.3672 | 2.3681 |
| 3 | 200 | 0.4 | 1000 | 171.44 | 175.64 | 172.86 | 42.50 | 40.08 | 41.28 | 75.21 | 77.18 | 75.97 | 76.12 | 0.5736 | 0.9935 |
| 4 | 300 | 0.4 | 1000 | 178.09 | 174.27 | 174.55 | 30.08 | 38.41 | 36.62 | 83.11 | 77.96 | 75.99 | 79.02 | 2.1226 | 3.6765 |
| 5 | 200 | 0.2 | 2000 | 168.47 | 173.71 | 172.15 | 53.54 | 49.23 | 50.44 | 68.22 | 71.66 | 72.22 | 70.70 | 1.2505 | 2.1659 |
| 6 | 300 | 0.2 | 2000 | 169.39 | 171.94 | 171.41 | 44.65 | 42.35 | 41.55 | 73.64 | 75.37 | 78.27 | 75.76 | 1.3507 | 2.3395 |
| 7 | 200 | 0.4 | 2000 | 173.78 | 173.34 | 173.91 | 30.69 | 34.20 | 34.59 | 82.34 | 80.27 | 77.72 | 80.11 | 1.3361 | 2.3142 |
| 8 | 300 | 0.4 | 2000 | 175.10 | 165.32 | 171.61 | 25.81 | 29.51 | 28.71 | 85.26 | 82.15 | 82.40 | 83.27 | 0.9976 | 1.7279 |
| 9 | 165.91 | 0.3 | 1500 | 173.05 | 174.92 | 171.26 | 45.08 | 41.77 | 43.98 | 73.95 | 76.12 | 72.89 | 74.32 | 0.9506 | 1.6465 |
| 10 | 334.09 | 0.3 | 1500 | 172.34 | 167.90 | 175.91 | 34.40 | 37.34 | 37.54 | 80.04 | 77.76 | 78.18 | 78.66 | 0.7006 | 1.2134 |
| 11 | 250 | 0.131821 | 1500 | 165.24 | 172.97 | 171.24 | 55.77 | 52.53 | 53.89 | 66.25 | 69.63 | 69.71 | 68.53 | 1.1402 | 1.9749 |
| 12 | 250 | 0.468179 | 1500 | 173.67 | 173.26 | 173.60 | 30.27 | 34.08 | 33.28 | 82.57 | 80.33 | 79.59 | 80.83 | 0.8958 | 1.5516 |
| 13 | 250 | 0.3 | 659.104 | 177.85 | 182.49 | 166.76 | 49.78 | 44.40 | 47.66 | 72.01 | 75.67 | 66.58 | 71.42 | 2.6406 | 4.5736 |
| 14 | 250 | 0.3 | 2340.9 | 174.30 | 165.96 | 172.35 | 36.36 | 40.51 | 39.76 | 79.14 | 75.59 | 76.06 | 76.93 | 1.1133 | 1.9283 |
| 15 | 250 | 0.3 | 1500 | 174.74 | 164.72 | 176.55 | 18.40 | 23.11 | 21.31 | 89.47 | 85.97 | 88.35 | 87.93 | 1.0320 | 1.7874 |
| 16 | 250 | 0.3 | 1500 | 179.28 | 166.22 | 165.80 | 16.96 | 19.63 | 19.83 | 90.54 | 88.19 | 85.39 | 88.04 | 1.4886 | 2.5783 |
| 17 | 250 | 0.3 | 1500 | 174.33 | 168.08 | 174.21 | 21.32 | 25.80 | 22.02 | 87.77 | 84.65 | 89.66 | 87.36 | 1.4607 | 2.5300 |
| 18 | 250 | 0.3 | 1500 | 178.52 | 161.39 | 177.64 | 16.87 | 28.55 | 23.75 | 90.55 | 82.31 | 87.03 | 86.63 | 2.3871 | 4.1345 |
| 19 | 250 | 0.3 | 1500 | 167.72 | 172.32 | 171.37 | 28.58 | 22.85 | 24.06 | 82.96 | 86.74 | 88.18 | 85.96 | 1.5565 | 2.6960 |
| 20 | 250 | 0.3 | 1500 | 182.20 | 171.59 | 170.36 | 17.09 | 24.76 | 21.96 | 90.62 | 85.57 | 85.14 | 87.11 | 1.7594 | 3.0473 |
